# A proposed definition of rare diseases for China: from the perspective of return on investment in new orphan drugs

**DOI:** 10.1186/s13023-015-0241-x

**Published:** 2015-03-07

**Authors:** Yazhou Cui, Jinxiang Han

**Affiliations:** Shandong Academy of Medical Science, Shandong Medical Biotechnological Center, Key Laboratory for Biotech Drugs of the Ministry of Health, Jinan, 250062 China

**Keywords:** Rare diseases, Definition, China, Orphan drug

## Abstract

A prevalence threshold to define rare diseases is needed for orphan drug designation. Here, we propose a bottom-up approach to defining rare diseases for China, based on the minimum number of patients needed for the industry to make a reasonable profit on a new drug. To obtain this patient population size, we considered three factors: (1) the industry research and development cost per new drug; (2) the sales per new drug to recoup its research and development costs and generate profit; (3) the highest affordable cost for one patient’s treatment in a given healthcare system. Using this model, we estimate that, with the current level of innovation in the pharmaceutical industry in China, between 300,000 and 500,000 patients could be a reference threshold to define rare diseases. Compared with other proposals, this evidence-based definition is more useful for designing rare diseases and orphan drug policies for China.

## Background

A rare disease is generally one with low prevalence but which is seriously debilitating or life-threatening. For most rare diseases, there are currently no curative or disease-modify treatments available. The definition for a rare disease differs between legislation and policies, and there is no single, widely accepted definition for rare diseases worldwide. Typically, the existing definitions include a criterion or cutoff of disease incidence or prevalence. For example, the United States (US) Food and Drug Administration (FDA) considers a rare disease to be one that affects <200,000 patients, and the criteria for the European Union (EU) and Japan are <250,000 patients (5 in 10,000) and <50,000 patients, respectively [1].

## Discussion

Public awareness of rare diseases has been increasing in China in the past decade. However, China has no legislation for the development of treatment for rare diseases, and gaps in the availability of orphan treatments for patients. The successful impact of legislation on orphan drug development in US and EU has evoked some experts and patient advocacy groups to propose that legislation for rare diseases and orphan drugs should be a high priority for China. China does not have an official definition of rare disease, and although the definition of the World Health Organization (prevalence between <0.65 – 1%) has been frequently used as a reference, this is not enough to provide a guide for the Chinese government to develop specific strategies for orphan drugs. For this, a prevalence threshold of rare diseases specific to China is necessary. In China, most of the rare diseases lack epidemiological data at the population level. Although the prevalence of several rare diseases has been extrapolated [2,3], actual patient numbers or the true prevalence of most rare diseases is often highly uncertain [4]. In 2010, some experts suggested that rare disease be defined by a prevalence of less than 1 in 500,000 or a neonatal morbidity of less than 1 in 10,000 [5]. However, this definition lacked supporting evidence.

Under normal marketing conditions, the pharmaceutical industry lacks incentive to commit to the high costs associated with developing a new drug for a disease with low prevalence. If the size of the patient population and the expected drug sales are inadequate to cover the return on investment in a new drug, such development would be financially unviable for the industry. In light of this, a rare disease could be defined as one that is not cost effective to treat [6]. Legislative incentives, for example, tax credits, research aids, simplification of the marketing authorization procedures, or extended market exclusivity can be introduced to make developing orphan drugs profitable.

Here, we suggest a simple approach to define rare diseases for China based on the return on investment in new orphan drugs. The core idea is to utilize the figure for the highest costs provided for one disease by a healthcare system to determine how many patients are needed to make a reasonable profit for developing a new drug. To obtain this patient population size in China, the following should be considered: (1) the research and development costs per new drug; (2) the sales of new drug needed to recoup its research and development costs and generate profit; (3) the highest amount payable to one patient in a given healthcare system.

Innovative drug research and development in China has progressed rapidly in the past decade. Although research and development costs are relatively low, the Chinese pharmaceutical industry lacks sufficient experience for innovative drug research and development and has a significantly high failure rate. The current total annual research and development spending in the life science-related industry as a whole on innovative drugs is about $5.0 billion [7], and an average of four innovative drugs have been approved for marketing per year in the past decade [8]. These innovative drugs only include chemical and biological drugs in class 1 (that is, not those included in traditional Chinese medicine and vaccines), which are defined by the Chinese State Food and Drug Administration (SFDA) as not being previously approved for marketing as a drug anywhere else in the world. Therefore, we estimate the research and development cost to develop a class 1 drug (including the costs of failures) in China to be about $1.2 billion. To recoup $1.2 billion in research and development costs and generate reasonable profit, sales per new drug should reach about $15–24 billion (based on research and development as 5–8% of annual sales in China [7]). Currently, the medical reimbursement ceiling in China is $50,000 annually per person, which means that at least 300,000–500,000 patients are needed per drug to guarantee the return on investment and ensure profit incentives for further research and investor support. Therefore, under the current level of innovation in the Chinese pharmaceutical industry, we suggest between 300,000–500,000 patients (about 2–4 out of 10,000, based on the Chinese population of 1.35 billion) as a reference threshold to define rare diseases. This prevalence threshold is greater than the threshold levels in the US and EU, but is comparative when the national population is considered. To our knowledge, this is the first evidence-based proposal for the definition of rare diseases.

China has a specific pharmaceutical market which is research and development focused (unique among developing countries), enabling the development of innovative orphan drugs. Additionally, and in contrast to practice in the US and EU, Chinese domestic research and development companies connect directly to the local market, not to the worldwide market. Most of the new drugs developed in China are used only or predominantly domestically, allowing global incentives and returns to be ignored in our model. Thus, our model determining the prevalence cutoff for rare diseases is only suitable for China, and could not be applied in an international, cross-border setting.

The limitations of this model are: (1) it was assumed that all patients would be diagnosed, treated and reimbursed by healthcare systems; (2) delays on return of investment are not considered. If these uncertain economic and environmental factors are included, the prevalence threshold might be higher than proposed. However, at least 300,000–500,000 could be a reference for the lower limit of prevalence to define rare diseases for China.

Currently, innovative drugs developed solely by domestic Chinese pharmaceutical companies are now all focused on common diseases. Without incentives, it is unlikely that domestic Chinese pharmaceutical companies will cover the costs of bringing an orphan drug to market. The Chinese government has not established a special program or funding plan for orphan drug research and development, but China is reforming its national health insurance and health care systems.

Finally, the prevalence threshold for the definition of rare diseases should be relatively fixed, though flexible, so that when the determining factors (costs of drug development, sales of new drugs and affordability of national healthcare systems) change dramatically, the definition could be re-evaluated. Moreover, the threshold suggested here is only a starting point, the final definition will depend on more detailed data on investment returns for new drugs in China.

## Summary

Based on this analysis, we propose that in future policies or legislation for rare diseases, orphan drugs treating seriously debilitating or life-threatening diseases with a prevalence between 300,000–500,000 should be a high priority in China.
